# Impact of Best-Fitted Control Selection on Effect Size: An Example in Functional GI Disorder Case–Control Studies

**DOI:** 10.3390/ijerph181910296

**Published:** 2021-09-29

**Authors:** Peyman Adibi, Shahram Agah, Hassan Doosti, Awat Feizi

**Affiliations:** 1 Isfahan Gastroenterology and Hepatology Research Center, Isfahan University of Medical Sciences, Isfahan 8174673461, Iran; adibi@med.mui.ac.ir; 2 Colorectal Research Center, Iran University of Medical Sciences, Tehran 1445613131, Iran; 3 Department of Mathematics and Statistics, Faculty of Science and Engineering, Macquarie University, Sydney, NSW 2109, Australia; 4 Department of Epidemiology and Biostatistics, School of Public Health, Isfahan University of Medical Sciences, Isfahan 8174673461, Iran; Awat_feiz@hlth.mui.ac.ir

**Keywords:** control selection, case–control studies, functional dyspepsia

## Abstract

Background: Effect sizes are the most useful quantities for communicating the practical significance of results and helping to facilitate cumulative science. We hypothesize that the selection of the best-fitted controls can significantly affect the estimated effect sizes in case–control studies. Therefore, we decided to exemplify and clarify this effect on effect size using a large data set. The objective of this study was to investigate the association among variables in functional gastrointestinal disorders (FGIDs) and mental health problems, common ailments that reduce the quality of life of a large proportion of the community worldwide. Method: In this methodological study, we constitute case and control groups in our study framework using the Epidemiology of Psychological, Alimentary Health and Nutrition (SEPAHAN) dataset of 4763 participants. We devised four definitions for control in this extensive database of FGID patients and analyzed the effect of these definitions on the odds ratio (OR): 1. conventional control: without target disorder/syndrome (sample size 4040); 2. without any positive criteria: criterion-free control (sample size 1053); 3. syndrome-free control: without any disorder/syndrome (sample size 847); 4. symptom-free control: without any symptoms (sample size 204). We considered a fixed case group that included 723 patients with a Rome III-based definition of functional dyspepsia. Psychological distress, anxiety, and depression were considered as dependent variables in the analysis. Logistic regression was used for association analysis, and the odds ratio and 95% confidence interval (95%CI) for OR were reported as the effect size. Results: The estimated ORs indicate that the strength of the association in the first case–control group is the lowest, and the fourth case–control group, including controls with completely asymptomatic people, is the highest. Ascending effect sizes were obtained in the conventional, criterion-free, syndrome-free, and symptom-free control groups. These results are consistent for all three psychological disorders, psychological distress, anxiety, and depression. Conclusions: This study shows that a precise definition of the control is mandatory in every case–control study and affects the estimated effect size. In clinical settings, the selection of symptomatic controls using the conventional definition could significantly diminish the effect size.

## 1. Introduction

Functional disorders are medical conditions that diminish the normal perception of body organs. They do not have a somatic etiology, and primarily remain undetected under examination or evaluations. They are usually multifactorial, and also show various ranges of manifestation [1]. Functional gastrointestinal disorder (FGID), one of the most common functional disorders, is a non-life-threatening disorder that negatively affects population health by impacting physical, social, and mental aspects. FGID is common in both developed and developing countries [2,3,4,5], markedly impairs quality of life, and significantly increases health costs in health care systems [3].

Like other functional disorders, FGID diagnosis is based on patient-reported measures that consist of ordinal time-based or severity-based variables with a defined cut-off point. In this respect, in 1990, the Rome criteria were introduced as a standard supporting better clinical diagnostic criteria of FGID [6]. From 1990 until now, three updates, including Rome II in 2000, Rome III in 2006, and Rome IV in 2016, have been published [4,5,6]. According to the Rome III criteria, FGID is categorized into six major fields for adults (the patients may either have one of these syndromes, or suffer from one or more symptoms). Functional dyspepsia (FD) is domain gastroduodenal (category b) among the six primary classes of FGID [6]. Because functional disorders have similar causative and interfering factors, people with a disorder often have a perceptual or third disorder.

Several factors and mechanisms such as stress and psychological disorders appear to be related to the development of functional gastrointestinal symptoms and disorders [2]. Moreover, several different treatments, such as antidepressants, acid-suppressing drugs [2], modulating eating behavior [7], anti-H. pylori therapy [8], and probiotics [9] have been considered.

### 1.1. Control Selection

Case–control studies are an efficient research method for investigating the risk factors of a disease. In a case–control study, the selection of controls is more complicated than the selection of cases [10]. The problems regarding finding an optimal comparison control group are among the most challenging issues in the design of case–control studies. The selection minimizes different types of bias while allowing greater generalizability of results and reliable effect size through a feasible recruitment approach. The appropriate choice of the control group and the excellent selection of cases on the basis of inclusion and exclusion criteria are crucial aspects to the design and execution of a logical case–control study [10]. Moreover, minimizing bias in control selection depends on suitable de-confounding. We should try to reduce selection bias, confounding bias, and information bias [11].

In diseases with subtle symptoms or mild manifestation, a satisfactory explanation of the case is required. In population-based studies, the control groups are selected from the same source as the cases. The control-to-case ratio could be significant; however, there is no noticeable improvement in accuracy when this ratio is increased beyond four [12].

The use of a sub-optimal control group can reduce the quality of a study [13]. Evidence suggests that design defects in all studies, including observational and experimental defects, can result in the overestimation or underestimation of the proposed effect sizes. Effect sizes are the most valuable quantities for communicating the practical significance of results and helping to facilitate cumulative science. Many studies have been conducted on the basis of maximizing participation and minimizing potential biases in case–control studies [14,15,16,17]. However, to the best of our current knowledge, there have been no studies examining the effects of different types of control groups on the estimated effect size, including odds ratio (OR), risk difference for categorical data, or mean difference for continuous variables. We emphasize that selection of the best-fitted controls can significantly affect the estimated effect sizes in case–control studies. Therefore, we decided to exemplify and clarify its effect on effect size in our large data set. We aimed to investigate the relationship of a variable in the scope of functional gastrointestinal disorders, such as FD and mental health problems, which are common ailments that reduce the quality of life of a large proportion of the community worldwide.

**Figure 1:**
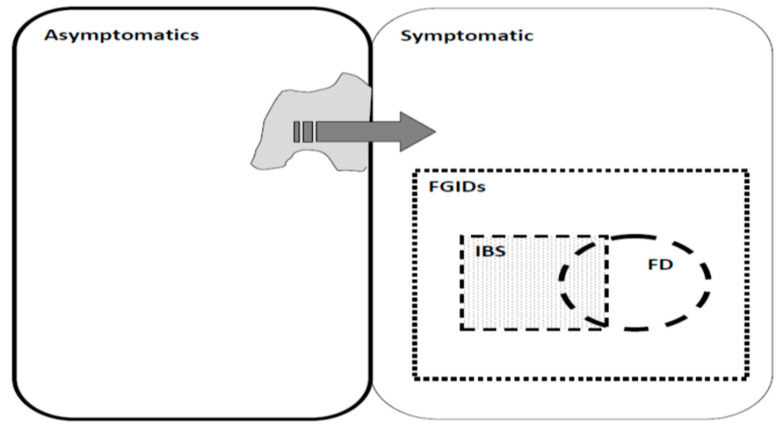
Control selection in Functional gastrointestinal disorders (FGID) with different approaches, Irritable bowel syndrome (IBS) and Functional dyspepsia (FD).

When approaching a population for evaluation of FD and its relation with psychological disorders using the Rome III criteria, we can consider different groups as controls (Figure 1).

During the selection of controls from a population, four options may be available (Table 1):

1. Conventional (without target disorder/syndrome based on Rome III criteria): those who may have a series of symptoms and even another syndrome, but did not fall into the case group according to the diagnostic means.
2. Criterion-free (without any positive criteria): those people whose report does not match the cut-off point of positive (partial symptomatic), although they may express some extent of illness.
3. Syndrome-free (without any disorder/syndrome): those people with some symptoms who could not be defined as having any other functional syndrome or case group definition (asyndromic control).
4. Symptom-free without any symptoms: those people who did not report any symptoms according to the diagnostic measures (asymptomatic controls); indeed, the case group criteria do not fit them.

Since more individuals who are in the control group may have some symptoms, but cannot regarded as patients because their symptoms are not sufficient to be considered as a syndrome. Another challenge is the time-based variance of the presence of complaints; a person may not report a minor transient symptom now, but may experience it two weeks later.

When using conventional control selection, the major problem is the possibility of confounding variables within both cases and controls. Moreover, some studies about FGID have similar common pathophysiology and risk factors, such as nutritional and psychological factors. These may induce and interfere with the generation and alteration of symptoms in all parts of the digestive system, forming a variety of syndromes. For example, some patients who have dyspepsia may experience some symptoms of irritable bowel syndrome (IBS). If the data analyst selects the control only by ruling out dyspepsia, many of the controls may have syndromes like IBS that could interfere in the analysis. The goal of our study was to evaluate the objective by evaluating the association between FD and the four above-mentioned scenarios with respect to control group selection and psychological disorders, including depression, anxiety, and psychological distress in a large adult sample. We show how each control group affects the strength of the estimated associations measured by the OR between variables.

### 1.2. Analysis Round

Table 1 summarises four analysis rounds in this study and explains how theoretical effect of different control groups impact on effect sizes (in current study odds ratio (OR)) in association analyses. In all analysis rounds, case is target disorder/syndrome positive and is defined those patients suffering from functional dyspepsia. Controls are one of the four defined groups. Table 1 lists our expectation of OR and p-value for each analysis rounds. In analysis round 1, OR will possibly be greater than one and corresponding p-value will be significant. In analysis round 2, it is more likely to have OR greater than one and the OR in the first round. We also expect smaller p-value in the round 2. That is expected that OR increases for rounds three and four and consequently corresponding p-values will decrease.

Corresponding syntaxs for definition of control groups are listed as follows:

- First control (Conventional); if subject had FD based on Rome-III, then FD = 1, else FD = 0
- Second control group (Criterion-Free); if subject never or sometimes experienced specific FGID symptom (xi) = 0, else xi = 1; compute sum = sum (symptom (xi) for all i = 1, …, 62. then FD = 1 if subject had sum > 0, else FD = 0 if sum = 0.
- Third control group (Syndrome-Free). If subject had no syndrome (xi), recode syndrome (xi) to 0, else = 1, compute sum = sum (syndrome (xi) for all i = 1, …, 17. then FD = 1 if sum > 0, else FD = 0 if sum = 0.
- Fourth control group (Symptom-Free). If subject reported symptom (xi) as never recode symptom (xi) to 0 else = 1, compute sum = sum (symptom (xi) for all i = 1, …, 62. then FD = 1 if sum > 0, else FD = 0 if sum = 0.

## 2. Materials and Methods

### 2.1. Study Design and Participants

In the study, we exemplify our study objective by using data from a real data set on a large sample of Iranian adults. We considered case and control groups from participants in the Study on the Epidemiology of Psychological, Alimentary Health and Nutrition (SEPAHAN) [18]. This cross-sectional study was conducted in two separate phases in Isfahan province, Iran, from April to May 2010. The primary aim of the SEPAHAN study was to examine the association of different lifestyle and psychological factors with gastrointestinal disorders. Multistage cluster and convenience sampling were used to select a group interested in participating in the study from among the 4 million people residing in Isfahan province. In the first phase, 10,087 pretested self-administered questionnaires were distributed to collect data on demographic and lifestyle variables, including dietary intakes, and 8691 completed questionnaires were returned (response rate: 86.16%). In the second phase, other questionnaires, designed to collect information on gastrointestinal, psychological, and somatoform symptoms, were distributed, and 6239 completed questionnaires were returned (response rate: 64.64%). After merging data from these two phases, complete information was available for 4763 people. Details about SEPAHAN have been reported previously [18]. Regional Bioethics Committee of IUMS (#189069, #189082, and #189086) approved the study protocol, and each participant provided a written informed consent form. In this secondary study, data from 4763 people were used for defining the case and control groups.

### 2.2. Procedures

#### 2.2.1. Case and Control Group Definition

The following steps were taken to pursue the current study objective, i.e., to determine the impact of different definitions of control groups on the estimated associations measures in epidemiological and clinical studies, specifically with respect to OR in the current study.

#### 2.2.2. Case Group

We considered a fixed case group in the current study. People who had functional dyspepsia based on ROM III criteria [19] and all defined control groups based on different definitions were compared in the proposed association analyses.

### 2.3. Functional Dyspepsia Assessment

FD was assessed as a dependent variable using a modified, reliable, and validated Persian version of the Rome III questionnaire to diagnose functional gastrointestinal disorders [18]. In the current study, FD was diagnosed on the basis of the participants’ answers to the following three questions: (a) ‘In the last three months, how often did you feel uncomfortably full after a regular-sized meal? (distressing postprandial fullness)’; (b) ‘In the last three months, how often were you unable to finish a regular-sized meal? (early satiation)’; and (c) ‘In the last three months, how often did you have pain or burning in the middle of your abdomen, above your belly button? (epigastric pain or epigastric burning)’. A person was considered to be an FD patient if they experienced one or more of the above-mentioned conditions at least often in the last three months [18]. Among the 4763 participants of the SEPAHAN study, 723 people suffered from FD, and these people were considered as the case group.

#### Control Groups

First control group (*n* = 4040): those people who did not suffer from FD based on Rome III criteria [4] were considered as a first control group in the current study; accordingly, while these people did not have FD, they may or may not have any other gastrointestinal disorders (conventional control).

Second control group (*n* = 1053): To construct the second control group, we dichotomized all clinical features, and those people who described experiencing all clinical symptoms either never or sometimes were considered to be partially symptom-free. Those that described experiencing clinical symptoms often or always were categorized into the second category. Based on this scenario, people with partially symptom-free were considered a control group of FD people (criterion-free: without any positive criteria).

Third control group (*n* = 847): this group was constructed on the basis of 17 FGID syndromes defined in the Rome III criteria [4]. The syndromes are binary variables, and people either had or did not have a specific syndrome. People with zero-sum were considered totally syndrome-free, and constituted the fourth counterpart control group for FD patients (syndrome-free: without any disorder/syndrome).

Fourth control group (*n* = 204): The FGIDs in Rome III include six major domains for adults: esophageal (category A), gastroduodenal (category B), bowel (category C), functional abdominal pain syndrome (category D), biliary (category E), and anorectal (category F). Each category site contains several disorders, each having relatively specific clinical features. In the Persian validated version, each clinical symptom has a 4-point Likert scale to assess the experienced gastrointestinal symptoms’ frequency (never/rarely, sometimes, often, always). To construct the fourth control group, we dichotomized all clinical features. Those people reported their experience of all clinical symptoms as never were considered to be totally symptom-free, and otherwise they were categorized in the fourth category. People that were totally symptom-free were regarded as a control group of FD people (symptom-free: without any symptoms).

On the basis of the above-mentioned variables, we finally constructed four binary variables; the first variable had two categories, including FD patients defined on the basis of Rome III (case group) vs. no-FD (conventional control group) in the framework of Rome III for FD evaluation; the second binary variable had two categories, including FD patients defined on the basis of Rome III (case group) vs. partially symptom-free people (second control group); the third binary variable had two categories, including FD patients defined on the basis of Rome III (case group) vs. totally syndrome-free people (third control group); and the fourth binary variable had two categories, including FD patients defined on the basis of Rome III vs. totally symptom-free people (fourth control group). We treated these variables as the dependent variable and compared the prevalence of three common psychological disorders (psychological distress, anxiety, and depression). We evaluated the prevalence of each psychological disorder separately between categories of four constructed binary variables for assessing the role of each specific control group.

### 2.4. Psychological Disorder Evaluation

We evaluated psychological distress as a potential FD contributor [19] using a validated Persian version of the general health questionnaire (GHQ)-12 [20]. The GHQ-12 contains 12 questions with a four-point rating scale, including “less than usual, no more than usual, rather more than usual, or much more than usual”. It estimates distress level using a bimodal scoring method (0-0-1-1). Accordingly, the two first answers were given a score of 0, and the two second answers were given a score of 1. Therefore, the possible score range would be 0–12, with higher scores indicating higher levels of psychological distress. On the basis of the mean of GHQ in the Iranian population, psychological distress was defined as a GHQ score ≥4. The Cronbach’s alpha coefficient for the Iranian version of GHQ-12 was 0.85 [20]. The alpha for the social dysfunction and psychological distress based on the split-half method was 0.77 and 0.76, respectively. A two-factor structure was obtained for the questionnaire, including social dysfunction and psychological distress that explained 48% of the overall observed variances [20].

Anxiety and depression in the SEPAHAN study were evaluated using a 14-item Iranian validated version of the Hospital, Anxiety and Depression Scale (HADS). HADS consists of two separate parts that measure the severity of anxiety and depression. In each section, there are seven items with a four-point Likert scale. Higher scores indicate a greater degree of anxiety or depression. The possible score ranged from 0 to 21 for both disorders. Scores of 8 or higher on either section were considered to indicate anxiety or depression, and scores of 7 or less were considered normal. The validity and reliability of the HADS questionnaire in the Iranian population have been validated. The internal consistency assessed by Cronbach’s alpha coefficient is 0.78 and 0.86 for the HADS anxiety and depression subscales, respectively. The validity was assessed by performing known groups comparison analysis, and showed satisfactory results, with both subscales being well-discriminated for patients with different medical conditions [21].

### 2.5. Statistical Analysis

Continuous and categorical variables are presented as mean (SD) and frequency (percentage). The prevalence of psychological disorders was compared between case and control groups using the chi-squared test. Additionally, we used univariate and multivariate-adjusted logistic regression for surveying the association of psychological disorders including depression, anxiety, and psychological distress as predictor variables and functional dyspepsia (in four scenarios, four different control groups) as the dependent variable. In univariate logistic regression, we only evaluated the association of FD with each psychological disorder. In multivariable logistic regression, we adjusted the confounding effects of age and gender. The strengths of associations were assessed by estimating OR and its 95%CI as the effect size. We evaluated the heterogeneity of estimated ORs by using Cochran Q chi-squared test. As a validation or sensitivity analysis for evaluating the consistency of results in different populations, we performed a subgroup analysis by gender. All aforementioned analyses were conducted separately in both genders. Statistical analyses were performed using SPSS version 25 (IBM: Armonk, NY, USA) and Statistical analysis for evaluatiing ORs heterogeneity was performed using R free statistical software version R-4.0.5 (R Core Team, 2021; The R Foundation, Vienna, Austria). The significance level was considered to be less than 0.05.

## 3. Results

Of the total of 4763 people in this study, 2106 (44.2%) were male, and 2657 (55.8%) were female. The mean (SD) age of the people in this study was 36.58 (8.093). The prevalence of FD was 723 (15.2%), and females had a higher prevalence rate 457 (17.2%) than males 266 (12.6%).

The demographic and psychological features of the study participants are presented in Table 2. The prevalence of depression, anxiety, and psychological distress were 1338 (28.8%), 654 (14%), and 1067 (23.1%), respectively. The prevalence of psychological disorders was significantly higher in women compared to men (*p* < 0.001). The prevalence of psychological disorders was significantly higher in FD patients compared to those in the four control groups. A descending trend in terms of the prevalence of psychological disorders can be seen from control group 1 to the fourth control group. These results indicate a lower prevalence of psychological disorders in purer controls.

**Figure 2:**
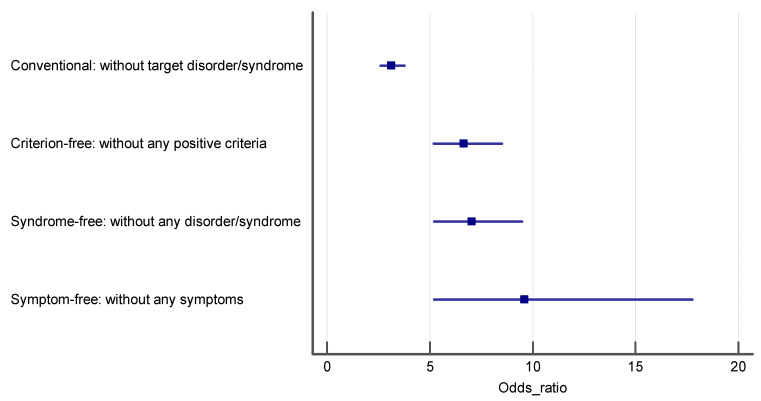
The estimated odds ratio for the association of psychological distress and FD in each control group.

**Figure 3:**
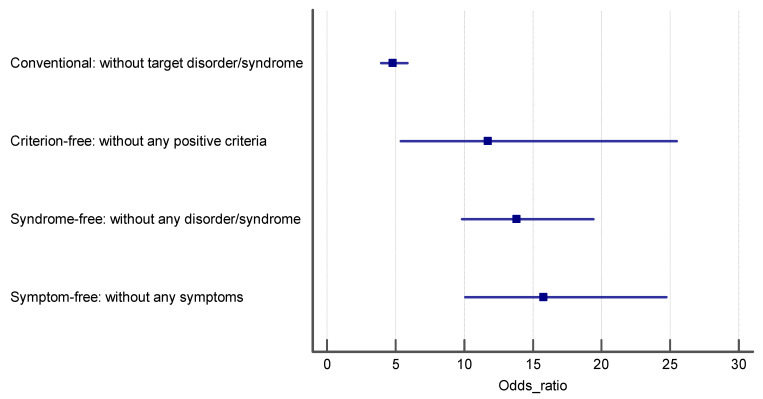
The estimated odds ratio for the association of anxiety and FD in each control group.

**Figure 4:**
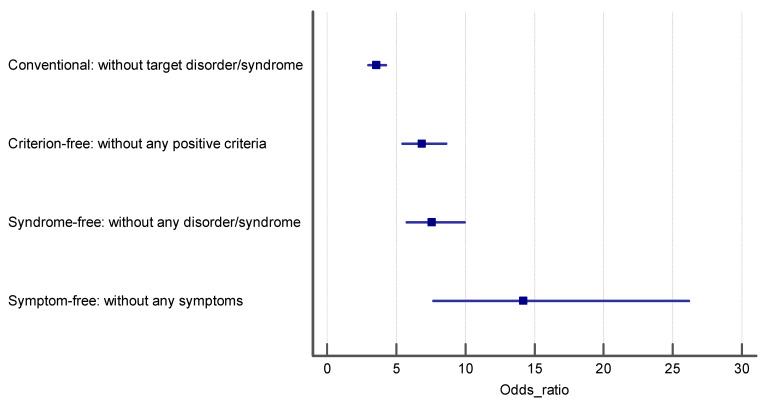
The estimated odds ratio for the association of depression and FD in each control group.

Table 3 represents the OR and 95%CI for the association of psychological disorders with functional dyspepsia in the total sample and separately for both genders. We constructed four logistic regression models, in crude and adjusted models, to investigate the association between psychological distress, anxiety, depression, and functional dyspepsia based on the four control groups. In the crude models, we only evaluated the association of psychological disorders with FD, while in the adjusted models, the potential confounding roles of age and gender were considered. The reported ORs in Table 3 confirm that the ORs in the first case–control group are the lowest, and the highest ORs were obtained in the fourth case–control group, which included controls with completely asymptomatic people. Ascending effect sizes were obtained for conventional, partially asymptomatic, syndrome-free and completely asymptomatic control groups, indicating that as the purity of controls increases, the intensity of the estimated effect size (OR) increases. The results of Cochran Q chi-squared test for heterogeneity of ORs for psychological distress (Q = 38.93 with degree of freedom—df = 3), anxiety (Q = 42.64, df = 3) and depression (Q = 29.51, df = 3) all are statistically significant at *p* < 0001. The ORs in the logistic regression models of control groups 2–4 were significantly higher than those obtained in the conventional control group (Q = 23.17, df = 1, *p* < 0.001, for psychological distress, Q = 27.45, df = 1, *p* < 0.001 for anxiety and Q = 20.45, df = 1, *p* < 0.001 for depression). The ORs in logistic regression models of control group 3 were significantly higher than those obtained in the conventional control group (Q = 20.58, df = 1, *p* < 0.001, for psychological distress, Q = 22.54, df = 1, *p* < 0.001 for anxiety and Q = 18.87, df = 1, *p* < 0.001 for depression). Finally, the estimated ORs in the logistic regression models of control group 4 were significantly higher than those obtained in the conventional control group except for anxiety (Q = 11.86, df = 1, *p* = 0.003, for psychological distress, Q = 2.58, df = 1, *p* < 0.34 for anxiety and Q = 9.55, df = 1, *p* = 0.006 for depression). Although ascending ORs were obtained for partially asymptomatic, syndrome-free and completely asymptomatic control groups, respectively, the estimated ORs were not significantly different (95%CI of ORs in each control group cover the point estimate of ORs of other control groups; Table 3, Figure 1, Figure 2 and Figure 3). These results are consistent for all three psychological disorders, psychological distress (Q = 1.19, df = 2, *p* = 0.55), anxiety (Q = 0.23, df = 2, *p* = 0.89), and depression (Q = 3.59, df = 2, *p* = 0.17). This means if the predictor variables are any of these three disorders, the ORs corresponding to the fourth control group will be the highest. In the sensitivity analysis, when we performed all of the association analyses separately in women and men, the same results were obtained (Table 3, Figure 2, Figure 3 and Figure 4).

## 4. Discussion

In this study, FD is an example of functional disorders used to explore the impact of selecting the control definition on the estimated effect size in a typical case–control study. Within the realm of functional disorders, there is an unclear border between health and disorder. This means that there is the possibility that independent variables may be shared between two or more functional disorders (we could see an overlap between several functional disorders). There are several methods for selecting control groups in case–control studies as a methodological paradigm.

The easiest method for selecting a control group in a case–control study is to choose participants from the population the study is examining that do not exhibit the case symptoms [21]. In this situation, there is a conceptual issue, that is, what are the symptoms? Should we consider only participants without any symptoms, or can we include those with a few symptoms, but no syndrome, as controls? On the other hand, in the definition of some disorders like FGID, we consider a criterion for the better classification of disorders and the discrimination of patients from non-patients. In these situations, many participants have another disease in their gastrointestinal system (such as common disorders like reflux disease) but which are not included as functional FGIDs.

To the best of our knowledge, this is the first study to point out the influence of changing the definition of the control group on the prevalence of functional disorders. Moreover, we estimate the effect of this change on the OR of predisposing factors like psychological distress on prevalence.

In this state-of-the-art analysis of our large database, we considered four different definitions for the control group, including “conventional: without target disorder/syndrome”, “syndrome-free: without any disorder/syndrome”, “criterion-free: without any positive criteria”, and “symptom-free: without any symptoms” (conventional control > criterion free > syndrome free > symptom-free). We considered the relationship between functional dyspepsia and psychological disorders, including anxiety and depression in both sexes, and with different control group definitions. We also measured the different percentage and OR for the prevalence of various psychological disorders when changing the definition of the control groups (Table 3).

We measured the OR and 95%CI for both sexes for the adjusted measurement in the second control group. According to this control definition, depression had the most significant OR for having FD.

Based on the third control group definition, anxiety had the largest OR for having FD.

We obtained the largest effect size values when the control group was at its purest, which was the fourth control group (symptom-free control group).

## 5. Conclusions

This study shows that a precise definition of the control is mandatory in every case–control study, and that the selection of different controls with different definitions could significantly affect the results of the study, including the relation of variables represented by various estimated effect sizes such as OR, hazard ratio, risk ratio, mean difference, correlation, and possibly many other statistical effect sizes. It is recommended that the type of control selection be reported in any functional disorder case–control study. This may be considered in future meta-analyses on series of observational studies.

## Figures and Tables

**Table 1:** Theoretical effect of different control group selection on impact size of the analysis in functional disorders.

Analysis Round	Case *: Target Disorder/Syndrome Positive	Control Type **	Odds Ratio (OR)	*p*-Value
1	N	Conventional: without target disorder/syndrome	Possibly greater than 1	Possibly significant
2	N	Criterion-free: without any positive criteria ***	Greater than 1 and first round	significant and smaller than first round
3	N	Syndrome-free: without any disorder/syndrome	Greater than 1 and second control group option	significant and smaller than second round
4	N	Symptom-free: without any symptom ^+^	Greatest (more than 1 and greater than third control group option)	significant and the smallest

* The definition and number of cases are similar in all four models: those who were defined as having syndrome according to a set of criteria. ** When working with a unique dataset, the control group 1 size > control group 2 size > control group 3 size > control group 4 size. *** A criterion is mainly defined as a dichotomous variable (0–1) based on a cut-off point. ^+^ Zero symptoms according to the questionnaire, i.e., those who never marked the time-frequency of a complaint. N: Number of cases.

**Table 2:** Demographical and psychological features of participants.

Variables	Psychological Distress (*n* = 4628)		Anxiety (*n* = 4657)		Depression (*n* = 4653)	
	Presence 1067 (23.1%)	Absence 3561 (76.9%)	Presence 654 (14%)	Absence 4003 (86%)	Presence 1338 (28.8%)	Absence 3315 (71.2%)
Sex						
Male	367 (18.1%)	1657 (81.9%)	204 (10%)	1841 (90%)	454 (22.2%)	1590 (77.8%)
Female	700 (26.9%)	1904 (73.1%)	450 (17.2%)	2162 (82.8%)	884 (33.9%)	1725 (66.1%)
Functional Dyspepsia						
Case N = 723	304 (42.9%)	404 (57.1%)	252 (35.5%)	458 (64.5%)	384 (54%)	327 (46%)
Control 1	763 (19.5%)	3157 (80.5%)	402 (10.2%)	3545 (89.8%)	954 (24.2%)	2988 (75.8%)
Control 2	148 (10.2%)	1300 (89.8%)	56 (3.8%)	1413 (96.2%)	203 (13.9%)	1262 (86.1%)
Control 3	74 (9.2%)	731 (90.8%)	24 (2.9%)	794 (97.1%)	103 (12.6%)	712 (87.4%)
Control 4	13 (6.7%)	181 (93.3%)	7 (3.6%)	186 (96.4%)	13 (6.7%)	180 (93.3%)

The discrepancies in sample size for analyzed variables are related to missing data. Controls 1–4 are conventional: without target disorder/syndrome, criterion-free: without any positive criteria, syndrome-free: without any disorder/syndrome, symptom-free: without any symptom, respectively.

**Table 3:** OR and 95% CI for the association of functional dyspepsia and psychological disorders.

Predictor Variables						
Dependent Variable Based on Different Control Selection	Psychological Distress (*n* = 4628)		Anxiety (*n* = 4657)		Depression (*n* = 4653)	
	OR	95%CI	OR	95%CI	OR	95%CI
Case–Control 1 (Total sample)						
Adjusted	3.128	2.607–3.755	4.803	3.930–5.871	3.556	2.973–4.254
Unadjusted	3.113	2.631–3.685	4.852	4.032–5.839	3.678	3.120–4.336
Case–Control 1 (male)						
Adjusted	4.238	3.079–5.833	5.859	4.024–8.529	4.488	3.289–6.122
Unadjusted	3.884	2.934–5.140	5.412	3.920–7.472	4.458	3.397–5.849
Case–Control 1 (female)						
Adjusted	2.713	2.175–3.383	4.468	3.525–5.663	3.183	2.560–3.957
Unadjusted	2.640	2.137–3.262	4.389	3.496–5.511	3.144	2.554–3.871
Case–Control 2 (Total sample)						
Adjusted	6.645	5.194–8.500	13.812	9.82–19.411	6.848	5.454–8.600
Unadjusted	6.610	5.273–8.285	13.883	10.202–18.893	7.300	5.923–8.998
Case–Control 2 (male)						
Adjusted	7.150	4.840–10.563	15.065	8.492–26.727	7.555	5.216–10.941
Unadjusted	6.736	4.758–9.537	13.397	8.236–21.793	7.751	5.584–10.758
Case–Control 2 (female)						
Adjusted	6.329	4.613–8.683	13.184	8.644–20.109	6.484	4.857–8.657
Unadjusted	6.165	4.559–8.337	12.813	8.570–19.157	6.403	4.866–8.427
Case–Control 3 (Total sample)						
Adjusted	7.039	5.223–9.488	15.785	10.077–24.724	7.564	5.753–9.945
Unadjusted	7.433	5.609–9.851	18.203	11.792–28.101	8.118	6.298–10.463
Case–Control 3 (male)						
Adjusted	6.422	4.049–10.187	12.966	6.415–26.208	8.417	5.343–13.259
Unadjusted	6.890	4.498–10.555	16.369	8.273–32.387	9.211	6.110–13.885
Case–Control 3 (female)						
Adjusted	7.615	5.128–11.308	18.452	10.232–33.277	7.307	5.171–10.323
Unadjusted	7.685	5.262–11.223	18.311	10.403–32.230	7.079	5.115–9.796
Case–Control 4 (Total sample)						
Adjusted	9.605	5.209–17.713	15.822	3.753–66.706	19.089	6.681–54.545
Unadjusted	10.477	5.853–18.752	20.670	4.970–85.961	19.052	7.506–48.359
Case–Control 4 (male)						
Adjusted	9.038	3.737–21.856	11.713	5.381–25.497	14.190	7.699–26.153
Unadjusted	9.097	4.060–20.385	14.620	6.769–31.579	16.26	9.087–29.095
Case–Control 4 (female)						
Adjusted	10.491	4.461–24.670	10.502	4.142–26.624	13.016	6.062–27.948
Unadjusted	11.588	4.963–27.059	11.096	4.418–27.869	13.591	6.429–28.734

## Data Availability

Data supporting reported results can be found, including links to publicly archived datasets analyzed or generated during the study.
